# HIV survey in Mozambique: analysis with simultaneous model in contrast to separate hierarchical models

**DOI:** 10.1186/s13690-020-00453-8

**Published:** 2020-07-31

**Authors:** Di Fang, Anqi Lang, Jeffrey R. Wilson

**Affiliations:** 1 Agribusiness, University of Arkansas, Fayetteville, USA; 2 School of Mathematics and Statistics, Arizona State University, Tempe, AZ USA; 3 Department of Economics, W. P. Carey School of Business, Computer Commons 465D, Arizona State University, Tempe, AZ 85287-9801 USA

**Keywords:** Shared-parameter model, Correlated, Intraclass correlation

## Abstract

**Background:**

The analysis of correlated responses obtained one at a time in survey data is not as informative or as useful as modeling them simultaneously. Simultaneous modeling allows for the opportunity to evaluate the system in a more pragmatic form rather than to allow for responses that assumedly originated in isolation.

**Methods:**

This research uses the Mozambique National Survey data to demonstrate the benefits of simultaneous modeling on blood test results, knowledge of HIV/AIDS, and awareness of an HIV/AIDS campaign. This simultaneous modeling also addresses the correlation inherent due to the hierarchical structure in the data collection.

**Results:**

Employment and self-perceived risk of HIV/AIDS have different impact on blood test, awareness of an HIV/AIDS campaign, and knowledge of HIV/AIDS when examined simultaneously as opposed to separate modeling.

**Conclusion:**

Simultaneous modeling of correlated responses improves the reliability of the estimates. More importantly, it provides an opportunity to engage in cost-saving decisions when designing future surveys and make better health policies.

## Background

It is common in national health research to use survey data to advance health policies. Survey results provide a measure of policy priorities. For example, the Demographic and Health Survey (DHS) is conducted in over 90 nations globally to obtain representative data on population health, nutrition, and HIV/AIDS. Data from these surveys are analyzed to identify trends and to advance global health research agendas and national programs and policies [1, 2].

National health surveys are often used to generate information that are critical in describing national and regional trends to identify gaps in knowledge. However, suboptimal analytic practices threaten the evidence base used for programmatic and policy decisions. Although national surveys capture multiple outcomes of interest, these outcomes are often modeled separately, thereby ignoring the correlation among outcomes or the interplay between outcomes. Also, the hierarchical design of national survey results in obtaining correlated observations are often omitted. This sort of omission leads to incorrect conclusions as incorrect standard errors are computed [3, 4]. The problem is best addressed with simultaneous modeling of responses while accounting for the hierarchical structure of the survey data.

Mozambique is an example of a nation in sub-Saharan Africa that is severely impacted by the HIV/AIDS epidemic. The disease is one of the single largest global health priorities of the past two decades, with $562.6 billion spent globally between 2000 and 2015 as reported by the Global Burden of Disease Health Financing Collaborator Network 2018. Innumerable analyses have characterized the HIV/AIDS epidemic and its drivers within and across contexts [5, 6]. Many of such decisions are made based on the Mozambique National Survey.

## Methods

### Mozambique survey data

This research utilizes a nationally representative, random sample of edited and cleaned from the Mozambique health data website. These data represent 270 clusters (primary units) distributed and sampled across Mozambique’s 11 provinces. The data consist of 6232 households (secondary units) eligible for sampling. Men and women aged 15–64 living in these households are at the observational level and are eligible to participate by giving blood samples. There are 9311 adult participants. These data are available to estimate the prevalence of HIV/AIDS in the general population and to determine the impact of factors.

### Outcomes of interest

This research concentrates on simultaneous modeling through the demonstration of three binary outcome measures of interest: blood test (positive or negative result), knowledge of HIV/AIDS (from community sources; a composite of a binary measure of participant awareness of HIV/AIDS from five sources: community meetings, school/teachers, conference in hospitals, community health workers, and church or mosque), and awareness of a campaign to combat HIV/AIDS (yes or no). These are binary outcome measures.

### Covariates

The covariates include the following demographics: continuous variables in age and years of education; and a categorical variable in gender, religion, marital status, employment in the past 12 months (not working, worked in the past year, currently working), family wealth index measured on 5-point ordinal scale (poorest, poorer, middle, rich, richest), and self-perceived risk of contracting HIV/AIDS (no risk, small risk, moderate risk, great risk, respondent HIV-infected). Binary factors include electricity in the household (*yes/no*) and received any support or social assistance (*yes/no*).

### Statistical model

#### Separate binary model

The modeling of binary outcomes often make use of a standard logistic regression model. The standard logistic regression model belongs to the group of generalized linear model. It operates on the assumption that the observations are independent. However, when analyzing hierarchical data the independence assumption is no longer acceptable, so the researcher uses a generalized linear mixed model over a generalized linear model. In the generalized linear mixed logistic regression model, one must account for the intraclass correlation at the different levels of the hierarchical structure, most commonly through the use of random effects.

The intraclass correlation coefficient (ICC) indicates how much of the total variation in the probability is accounted for by the hierarchical level of the data. However, in fitting binary models, it often appears that there is no error at the lowest level of the hierarchy (level-1), but that incorrect assumption still must be addressed. Therefore, a slight modification is needed to calculate the ICC. This modification assumes the dichotomous outcome comes from an unknown latent continuous variable with a level-1 residual that follows a logistic distribution with a mean of 0 and a variance of 3.29. Therefore, 3.29 is used as our level-1 error variance in calculating the ICC [7]. In these data, there are three levels, thus two random effects are identified. One random effect represents household effects and the other random effect represents the cluster effects [4]. Then, 3.29 represents variance of the residuals at the observational level (resident) [8].

Thus, for modeling one binary response, a generalized linear mixed model with the clusters and the households incorporated as random effects to model the contribution due to households and clusters respectively is

1 $$ \log \left[\frac{{\mathrm{p}}_{\mathrm{ihc}}}{1-{\mathrm{p}}_{\mathrm{ihc}}}\right]={\upbeta}_0+{\upbeta}_1{\mathrm{X}}_{1\mathrm{hc}}+\cdots \cdots \cdots \cdots \cdots +{\upbeta}_{11}{\mathrm{X}}_{11\mathrm{hc}}+{\mathrm{u}}_{\mathrm{oc}}+{\mathrm{u}}_{\mathrm{ohc}} $$

where p_ihc_ is the probability of a favorable outcome for the i^th^ resident within the h^th^ household within the c^th^ cluster, β_i_ is the regression coefficient associated with the predictor X_ihc_ for i = 1,2, …, n_hc_; are the covariates associated with the h^th^ household within that c^th^ cluster, and a random effect household *h* =1, 2, …, n_c_; and cluster c = 1, …, n; the random intercept u_oc_ measures the unobserved variance attributable to the c^th^ cluster, the random intercept u_ohc_ measures differences of household level h within cluster c *.* These two random effects are assumed to be normally distributed with ${\mathrm{u}}_{\mathrm{oc}}\sim \mathcal{N}\left(0,{\upsigma^2}_{{\mathrm{u}}_{\mathrm{c}}}\right)$ and ${\mathrm{u}}_{\mathrm{ohc}}\sim \mathcal{N}\left(0,{\upsigma^2}_{{\mathrm{u}}_{\mathrm{hc}}}\right)$. Further, this research assumes that the covariance of the random effects, ${\sigma}_{{\mathrm{u}}_{\mathrm{oc}},{\mathrm{u}}_{\mathrm{ohc}}}$ is zero. Households as random effects represent the differences in the residents’ responses attributable to households, but were not captured by any of the covariates at the household level. Similarly, clusters as random effects represent the differences in the residents’ responses attributable to the clusters, but were not captured by any of the covariates at the cluster level.

#### Simultaneous models

In public health research, it is common to find subjects providing information on a cadre of health responses with a set of covariates. However, the correlation among these responses is helpful to public health officials and decision makers. The identification of the overlap helps with distribution of resources and helps avoid duplication. Thus, it is advantageous to have simultaneous modeling.

This research demonstrates use of three simultaneous binary outcomes Y_1ihc_, Y_2ihc_, and Y_3ihc_ denoting the i^th^ individual on the h^th^ household member of the c^th^ cluster for outcomes *q*= 1, 2, and 3 for *h* = 1, …. . *n*_*c*_, and *c* = 1, …..270. A simultaneous model of these binary outcomes f(Y_1hc_, Y_2hc_, Y_3hc_) consists of a shared-parameter that measures the correlation among the outcomes [8]. For *q* = 1, the response Y_1ihc_ for blood test follow a Bernoulli distribution with mean p_1hc_ and random effects u_oc_ for clusters and random effects u_ohc_ for households thus,

$$ \mathrm{logit}\ \left({\mathrm{p}}_{1\mathrm{hc}}\right)={\upbeta_{00}}^{\mathrm{q}=1}+{\upbeta_1}^{\mathrm{q}=1}{\mathrm{x}}_{1\mathrm{hc}}+\dots +{\upbeta_{\mathrm{p}}}^{\mathrm{q}=1}{\mathrm{x}}_{\mathrm{p}\mathrm{hc}}++{{\mathrm{u}}_{\mathrm{oc}}}^{\mathrm{q}=1}+{{\mathrm{u}}_{\mathrm{ohc}}}^{\mathrm{q}=1}. $$

Similarly, models are available for heard of HIV/AIDS campaign [2] (q = 2) and heard of HIV/AIDS responses (q = 3). The joint modeling of these three outcomes has a vector of random effects **u** distributed as normal with mean vector **0** and covariance matrix, ${\mathrm{D}}_{\mathrm{H}\cap \mathrm{C}}^{123}$, such that the random effects for levels in the hierarchy is

$$ \mathbf{u}=\left(\begin{array}{c}{{\mathrm{u}}_{\mathrm{oc}}}^1\\ {}{{\mathrm{u}}_{\mathrm{ohc}}}^1\\ {}\begin{array}{c}{{\mathrm{u}}_{\mathrm{oc}}}^2\\ {}{{\mathrm{u}}_{\mathrm{ohc}}}^2\\ {}\begin{array}{c}{{\mathrm{u}}_{\mathrm{oc}}}^3\\ {}{{\mathrm{u}}_{\mathrm{ohc}}}^3\end{array}\end{array}\end{array}\right)\sim \mathbb{N}\left[\left(\begin{array}{c}0\\ {}0\\ {}\begin{array}{c}0\\ {}0\\ {}\begin{array}{c}0\\ {}0\end{array}\end{array}\end{array}\right),\kern0.5em {\mathbf{D}}_{\mathrm{H}\cap \mathrm{C}}^{123}=\left(\begin{array}{c}{\mathrm{d}}_1^1\\ {}0\\ {}\begin{array}{c}{\mathrm{d}}_1^{12}\\ {}\begin{array}{c}0\\ {}{\mathrm{d}}_1^{13}\\ {}0\end{array}\end{array}\end{array}\kern0.5em \begin{array}{c}\begin{array}{c}0\\ {}{\mathrm{d}}_2^1\\ {}0\end{array}\\ {}{\mathrm{d}}_2^{12}\\ {}\begin{array}{c}0\\ {}{\mathrm{d}}_2^{13}\end{array}\end{array}\kern0.5em \begin{array}{ccc}\begin{array}{c}\begin{array}{c}{\mathrm{d}}_1^{12}\\ {}0\\ {}{\mathrm{d}}_1^2\end{array}\\ {}0\\ {}\begin{array}{c}{\mathrm{d}}_1^{23}\\ {}0\end{array}\end{array}& \begin{array}{c}0\\ {}\begin{array}{c}{\mathrm{d}}_2^{12}\\ {}0\\ {}{\mathrm{d}}_2^2\end{array}\\ {}\begin{array}{c}0\\ {}{\mathrm{d}}_2^{23}\end{array}\end{array}& \begin{array}{cc}\begin{array}{c}{\mathrm{d}}_1^{13}\\ {}\begin{array}{c}0\\ {}{\mathrm{d}}_1^{23}\\ {}0\end{array}\\ {}\begin{array}{c}{\mathrm{d}}_1^3\\ {}0\end{array}\end{array}& \begin{array}{c}0\\ {}\begin{array}{c}{\mathrm{d}}_2^{13}\\ {}0\\ {}{\mathrm{d}}_2^{23}\end{array}\\ {}\begin{array}{c}0\\ {}{\mathrm{d}}_2^3\end{array}\end{array}\end{array}\end{array}\right)\right]. $$

This ${\mathrm{D}}_{\mathrm{H}\cap \mathrm{C}}^{123}$ covariance matrix contains the two random effects operating at different levels for the same response are independent, $$ \left(\begin{array}{cc}{\mathrm{d}}_1^{\mathrm{q}}& 0\\ {}0& {\mathrm{d}}_2^{\mathrm{q}}\end{array}\right) $$ for *q* = 1, 2, 3. In this scenario, there is no correlation among the random effects for a given outcome. However, there is correlation among the random effects across the different responses at the same level but not at different levels, for example $$ \left(\begin{array}{cc}{\mathrm{d}}_1^{13}& 0\\ {}0& {\mathrm{d}}_2^{13}\end{array}\right) $$. If the covariance ${\mathrm{d}}_1^{qp}=0$ then they are uncorrelated, and the resulting model is equivalent to modeling the three outcomes separately [9, 10]. The result of a test that [${\mathrm{d}}_1^{12},{\mathrm{d}}_2^{12},{\mathrm{d}}_1^{13},{\mathrm{d}}_2^{13},{\mathrm{d}}_1^{23},{\mathrm{d}}_2^{23}$] are simultaneously zero determines if one needs the simultaneous model or if one will be satisfied with separate models.

Consider the model $\mathrm{logit}\ \left({\hat{\mathrm{p}}}_{\mathrm{qhc}}\right)$, for q = 1, 2, 3. Let the vector ${\mathrm{W}}_{\mathrm{Yhc}}^{\mathrm{b}}$ denotes the difference between the observed values and the model values, such that

$$ {\mathrm{W}}_{\mathrm{Y}}^{\mathrm{b}}={\mathrm{N}}^{-1}\sum \limits_{\mathrm{c}=1}^{{\mathrm{n}}_{\mathrm{c}}}\sum \limits_{\mathrm{h}=1}^{{\mathrm{n}}_{\mathrm{h}\mathrm{c}}}\left({\mathrm{W}}_{\mathrm{Y}\mathrm{hc}}^{\mathrm{b}}\right){\left({\mathrm{W}}_{\mathrm{Y}\mathrm{hc}}^{\mathrm{b}}\right)}^{\mathrm{T}}. $$

The joint log-likelihood is

$$ \mathrm{Log}\ {\mathrm{L}}^{123}=\prod \limits_{b=1}^3\left\{\left[{\mathrm{C}\mathrm{ov}}^{-1}{\mathrm{W}}_{\mathrm{Y}}^{\mathrm{b}}\right]-\frac{1}{2}\sum \limits_{\mathrm{c}=1}^{\mathrm{C}}\sum \limits_{\mathrm{h}=1}^{{\mathrm{h}}_{\mathrm{c}}}\log \left|{\mathrm{D}}_{\mathrm{H}\cap \mathrm{C}}^{123}\right|-\frac{1}{2}\sum \limits_{\mathrm{c}=1}^{\mathrm{C}}{\mathbf{u}}_{\mathrm{c}}^{\mathrm{T}}{{\mathrm{D}}_{\mathrm{H}\cap \mathrm{C}}^{123}}^{-1}{\mathbf{u}}_{\mathrm{c}}\right\} $$

which achieves maximum estimator

$$ \hat{\boldsymbol{\upbeta}}={\left(\sum \limits_{\mathrm{c}}^{\mathrm{C}}\sum \limits_{\mathrm{h}}^{{\mathrm{H}}_{\mathrm{c}}}{\mathrm{X}}_{\mathrm{h}\mathrm{c}}^{\mathrm{T}}{{\mathrm{D}}_{\mathrm{H}\cap \mathrm{C}}^{123}}^{-1}{\mathrm{X}}_{\mathrm{h}\mathrm{c}}\right)}^{-1}\left(\sum \limits_{\mathrm{c}}^{\mathrm{C}}\sum \limits_{\mathrm{h}}^{{\mathrm{H}}_{\mathrm{c}}}{\mathrm{X}}_{\mathrm{h}\mathrm{c}}^{\mathrm{T}}{{\mathrm{D}}_{\mathrm{H}\cap \mathrm{C}}^{123}}^{-1}\left(\hat{{\mathrm{w}}_{\mathrm{Yhci}}^{\mathrm{b}}}\right)\right) $$

where

$$ \hat{{\mathrm{D}}_{\mathrm{H}\cap \mathrm{C}}^{123}}=\frac{1}{\mathrm{N}}{\sum}_{\mathrm{c}}^{\mathrm{C}}{\sum}_{\mathrm{h}}^{{\mathrm{H}}_{\mathrm{c}}}{\mathrm{w}}_{\mathrm{Yhci}}^{\mathrm{b}}{{\mathrm{w}}_{\mathrm{Yhci}}^{\mathrm{b}}}^{\prime }. $$

Through, the use of a modification of the expectation-maximization (EM) algorithm, the researcher is able to obtain maximum-likelihood estimates for model parameters when there is unobserved (hidden) latent variables.

The maximum likelihood estimates for the correlated logistic regression model is obtained [11]. The iteration process in the EM algorithm context provides convergence to the true ML estimates [12]. It is an iterative way to approximate the maximum likelihood function.

This research presents simultaneous generalized linear mixed models for binary responses (knowledge of HIV/AIDS, awareness of an HIV/AIDS campaign, and blood testing for HIV/AIDS) using a shared joint random effects. This research uses these survey data to demonstrate the advantages of simultaneous modeling of these responses. These data are obtained based on a hierarchical structure. The SAS procedure PROC QLIM, among other models, fit simultaneous binary models. This procedure is designed to analyze mainly cross-sectional data.

## Results

The survey data contained 58% of respondents who are female and nearly 70% of respondents who are married or living with a partner. The average age of the respondents is 31 years, and the average years of education is three. Of the respondents, 54.08% are Catholic or Muslim. Approximately 25% households have electricity. Approximately 16% of the respondents did not work in the last 12 months. About 30.7% of the respondents are classified into the richest category, and 12.2% of the respondents are classified into the poorest category. About 31% of the respondents perceived that they had no risk of contracting HIV/AIDS. The blood tests reveal 13.4% of respondents are HIV-infected. There are 77.2% of the respondents aware of HIV from community organizations and other institutes (schools, hospitals, religious institutions), and about 55% of respondents are aware of a campaign to combat HIV/AIDS. These results are summarized in Table 1.

**Table 1:** Overview of demographic characteristics of the sample and results for the three endpoints (HIV blood test, awareness of HIV/AIDS, awareness about HIV/AIDS campaign)

Variable	Frequency (%) ***n*** = 9331
Female	5402 (58.0%)
Age (years ± SD)	31 ± 12.5
**Marital status**	
Never married	1430 (15.4%)
Married	1035 (11.1%)
Living together	5447 (58.5%)
Widowed	527 (5.7%)
Divorced	168 (1.8%)
Not living together	704 (7.6%)
**Religion**	
Catholic	2766 (29.7%)
Islamic	2269 (24.4%)
Zionist	877 (9.4%)
Evangelical/Pentecostal	1600 (17.2%)
Anglican	100 (1.1%)
No religion	1219 (13.1%)
Other	480 (5.2%)
**Education,** years ± SD (min, max)	3 ± 3.69 (0, 19)
**Employment**	
Not employed	1513 (16.3%)
Employed in the past year	1146 (12.3%)
Currently working	6652 (71.4%)
**Support**	
No	8905
Yes	390
Don’t know	16
**Self-perceived risk of HIV/AIDS**	
No risk	2840
Small risk	1957
Moderate risk	868
Great risk	869
Respondent HIV-infected	96
Don’t know	**=2681**
**Measures of Wealth**	
Electricity in household	2353 (25.3%)
Refrigerator in household	1456 (15.6%)
**Wealth index**	
Poorest	1133 (12.2%)
Poorer	1525 (16.4%)
Middle	1760 (18.9%)
Richer	2037 (21.9%)
Richest	2856 (30.7%)
Blood Test results HIV-infected	1246 (13.4%)
Aware of HIV/AIDS	7191 (77.2%)
Aware of HIV/AIDS Campaigns	5110 (54.9%)

Total respondents were less than 9331 for social support (*n* = 9295) and for self-perceived risk of HIV/AIDS (*n* = 6630) due to selection of “I don’t know”

There are no respondents in the survey who tested positive and did not hear about HIV/AIDS, but heard about the campaign. In addition, there are no respondents who tested negative and did not hear about the disease, but heard about the campaign, as shown in Table 2.

**Table 2:** Simultaneous responses to three binary responses

HIV Blood test	Awareness of HIV/AIDS	Awareness of HIV/AIDS campaign	Frequency	%
0	0	0	1910	20.51
0	1	0	1814	19.48
0	1	1	4341	46.62
1	0	0	210	2.26
1	1	0	267	2.87
1	1	1	769	8.26

The data are collected in an hierarchical structure. Respondents are nested within households and households are nested within clusters. The correlation due to this structure, households and clusters, are considered as random effects. The variances of the random effects at the household level and at the cluster levels are shown in Table 3. The estimates suggest that the variance of the random effects due to clusters are significant (blood test, aware of HIV/AIDS, and aware of campaign) and too large to ignore in any model [4]. The variance of the random household effects are significant in measuring the blood test, but not significant when modeling for awareness of HIV and awareness of campaigns.

**Table 3:** Random effects for each response at cluster and household

HIV Blood test	Effects	Estimate	Standard Error	***p***-value
Intercept	cluster	0.394	0.079	<.0001
Intercept	HH (cluster)	0.385	0.114	0.0004
**Aware of HIV/AIDS**				
Intercept	cluster	0.696	0.095	<.0001
Intercept	HH (cluster)	0.039	0.083	0.318
**Aware of HIV/AIDS campaign**				
Intercept	cluster	0.851	0.104	<.0001
Intercept	HH (cluster)	0.067	0.073	0.179

### Simultaneous hierarchical logistic models

The simultaneous modeling of the three binary outcomes provides an opportunity to address interplay among the responses. The estimates for this simultaneous model of these three binary outcomes (knowledge of HIV/AIDS, awareness of HIV/AIDS campaign, and blood testing for HIV/AIDS) are given in Table 4.

**Table 4:** Comparison of regression coefficients and p-values for the separate and simultaneous logistic models of the three outcomes on demographic characteristics

		HIV Blood test				Awareness of HIV/AIDS campaign				Awareness of HIV/AIDS campaign			
		Separate		Joint		Separate		Joint		Separate		Joint	
	Variable	coeff	*p*-value	coeff	*p*-value	coeff	*p*-value	coeff	*p*-value	coeff	*p*-value	coeff	*p*-value
	*Electricity*	− 0.297	0.036	− 0.181	0.007	0.471	0.004	0.247	0.001	0.877	<.0001	0.445	<.0001
Wealth	Poorer	0.25	0.223	0.119	0.2	−0.029	0.809	− 0.072	0.244	0.017	0.889	0.05	0.344
	Middle	0.332	0.106	0.139	0.127	0.038	0.759	0.054	0.38	0.245	0.049	0.194	0
	Richer	0.89	<.001	0.525	<.001	0.179	0.193	0.126	0.051	0.443	0.001	0.271	<.001
	Richest	1.225	<.001	0.693	<.001	0.449	0.015	0.251	0.002	0.618	0	0.398	<.001
Education	Education (years)	0.02	0.147	0.008	0.247	0.149	<.0001	0.077	<.001	0.173	<.001	0.09	<.001
Age	Age (years)	−0.003	0.507	− 0.002	0.366	− 0.005	0.106	− 0.003	0.065	− 0.005	0.092	−0.003	0.055
Gender	Male v. Female	−0.127	0.179	−0.053	0.264	−0.055	0.48	0.025	0.552	0.676	<.001	0.324	<.0001
Religion	Catholic	−0.173	0.378	−0.082	0.39	−0.304	0.166	−0.289	0.007	−0.317	0.074	−0.212	0.004
	Islamic	−0.052	0.787	0.016	0.866	−0.278	0.204	−0.252	0.02	−0.422	0.016	−0.278	0
	Zion	0.224	0.302	0.163	0.135	−0.318	0.183	−0.29	0.015	−0.385	0.05	−0.291	0.001
	Pentecostal	−0.501	0.028	−0.222	0.037	−0.439	0.061	−0.412	0	−0.22	0.264	−0.136	0.088
	Anglican	−0.098	0.82	0.029	0.895	−1.293	0.001	−0.568	0.006	−1.091	0.003	−0.512	0.003
	No religion	0.141	0.511	0.114	0.281	−0.713	0.002	−0.59	<.001	−0.542	0.004	−0.359	<.001
Marital Status	Married	0.893	<.001	0.448	<.001	−0.155	0.354	− 0.045	0.599	−0.431	0.003	−0.147	0.034
	Living together	0.947	<.001	0.485	<.001	−0.163	0.223	−0.091	0.175	−0.262	0.021	−0.123	0.022
	Widowed	1.84	<.001	0.991	<.001	−0.407	0.043	−0.134	0.208	−0.268	0.142	−0.085	0.333
	Divorced	1.645	<.001	0.876	<.001	−0.454	0.083	−0.219	0.119	−0.301	0.229	−0.093	0.453
	Not living together	1.692	<.001	0.88	<.001	−0.275	0.136	−0.085	0.367	−0.265	0.095	−0.122	0.106
Employment	Employment: In the past year	0.138	0.416	0.064	0.452	−0.435	0.004	−0.211	0.006	− 0.16	0.237	− 0.136	0.027
	Currently working	0.062	0.636	0.002	0.978	−0.204	0.1	−0.08	0.195	0.004	0.971	0.023	0.626
Support	Support	0.112	0.594	0.051	0.618	0.565	0.002	0.184	0.039	0.096	0.541	−0.053	0.446
Self-perceived risk	Small risk	0.271	0.013	0.151	0.004	0.426	<.0001	0.221	<.001	0.443	<.001	0.248	<.001
	Moderate risk	0.453	0.001	0.227	0.001	0.141	0.235	0.151	0.014	0.322	0.003	0.23	<.001
	Great risk	0.653	<.001	0.356	<.001	0.413	0.002	0.275	<.001	0.517	<.0001	0.308	<.001
	Respondent HIV+	3.637	<.001	2.182	<.001	1.172	0.006	0.674	0.001	1.269	<.0001	0.717	<.001
	Intercept	−3.873	<.001	−2.104	<.001	1.432	<.001	0.866	<.001	−0.565	0.018	−0.37	0

The model shows that having electricity in the house increases the likelihood of hearing about the HIV/AIDS campaign and decreases the HIV-infected rate (*p* < 0.0074). Wealthiest Mozambicans are more likely to have a positive blood test, knowledge of HIV/AIDS, and awareness of HIV/AIDS campaign in all models (*p* < 0.0024). Respondents with more years of education are more likely to be aware of HIV/AIDS campaign (*p* < 0.001). Respondents who perceived any risk (small, moderate, great) are more likely to have HIV-infected test results compared to those perceiving no risk (*p* < 0.001). Those residents who are married or living together are more likely to be HIV-infected (*p* < 0.001). Males are more likely to hear about HIV/AIDS campaign (*p* < 0.001). Support or social assistance is a significant factor only for knowledge of HIV/AIDS (*p* = 0.039). Marital status has no effect on knowledge but has an impact on blood test and awareness (*p* < 0.001 and (*p*-0.034) respectfully. Risk of AIDS and richer residents are significant for all three responses. Similar covariates are significant in modeling awareness of campaigns to combat HIV/AIDS and for modeling awareness of HIV/AIDS (Table 4).

There are marked difference in separate modeling of these responses versus simultaneous modeling the responses. The simultaneous modeling accounts for the other responses in determining the impact of a covariate on a particular response. A separate response model is compared to the simultaneous model, as shown in Table 4. It shows *p*-values (0.165 v 0.007 and 0.074 v 0.004) for knowledge of HIV/AIDS and awareness of HIV/AIDS campaigns, respectively. The impact of employed residents [in the past year] on awareness of HIV/AIDS is affected by the association between knowledge of HIV/AIDS and awareness of HIV/AIDS campaigns. The separate response model and the simultaneous model for employment for awareness of campaign showed *p*-values of (0.2368 v 0.0267). The impact of [moderate risk] on knowledge of HIV/AIDS is affected by the association between knowledge of HIV/AIDS and awareness of HIV/AIDS campaign. The single model versus a simultaneous model showed *p*-values (0.235 v 0.014 [self-perceived risk]) for knowledge of HIV/AIDS.

## Conclusion

The survey data are correlated due to the hierarchical structure of the data. Statistical methods for the analysis of correlated data have become more accessible as statistical programs include the opportunity to use such models. The fit of correlated data with a generalized linear mixed model is common. However, it is important to note the analysis of correlated data does not have the same interpretation as when the data are assumed independent in its analysis. The analysis of correlated data with random effects are referred to as subject-specific model.

Modeling simultaneous responses allows researchers to address correlation and explain the interplay. Such information results in cost saving measures in the design of future surveys. The advantage of simultaneous modeling lies with its ability to address one response while controlling for another. It is typical in survey data to have the respondents provide responses to a series of outcomes. More importantly, the simultaneous modeling of responses on hierarchical data provides policymakers and researchers with results on which to base allocation of resources at a time when funding is a scarce commodity.

Researchers are often faced with data with complicated structure but often choose to forgo complex models and rely on two-at-a-time modeling, one response and one covariate, with independent observations. However, there are multivariable methods [one response and several covariates] based on independent observations. For analysis, when the observations are not independent, a correlated model is necessary to identify the pattern of association. Such a model provides larger standard errors, which affects the significance of the covariates.

The analysis of the 2018 Mozambique survey data, like most survey data, present simultaneous responses [5, 6]. Modeling simultaneous responses allows for the interpretation of interplay, which can lead to cost saving in future surveys. This approach is unique in that it addresses simultaneously the factors and the extra variation, as well as the interplay usually seen in survey data [13].

## Supplementary information

**Additional file 1.**

## Data Availability

The datasets during and/or analyzed during the current study available from the corresponding author on reasonable request.

## Abbreviations

DHS: Demographic and Health Survey
HIV: Human immunodeficiency virus
AIDS: Acquired immunodeficiency syndrome
