# Supplementary material for: HIV survey in Mozambique: analysis with simultaneous model in contrast to separate hierarchical models
Source: Arch Public Health. 2020 Jul 31;78:70. doi: 10.1186/s13690-020-00453-8 (PMC7395358; doi:10.1186/s13690-020-00453-8)
Supplement: Supplementary file 1 — Additional file 1. [file 13690_2020_453_MOESM1_ESM.docx]

**Appendix**

*Three levels;

PROC GLIMMIX data=Mozambique_new ;

class HH cluster electricity refrigerator Wealthindex gender Religion maritalstatus Respondentworked receivesupport riskofAIDS;

model bloodtest(event='1')=electricity refrigerator Wealthindex yearsofeducation

currentage gender Religion maritalstatus Respondentworked receivesupport riskofAIDS/DIST=BINARY LINK=LOGIT DDFM=BW SOLUTION OR;

random intercept/subject=cluster solution TYPE=VC;

random intercept/subject=HH(cluster) TYPE=VC;

covtest/WALD;

run;

*The output is in SAS Output_qlimtest file and the second qlim procedure;

proc qlim data=Mozambique_new method=newrap;

class HV206 HV209 HV270 AIDSEX V130 V501 V731 S122 S423;

endogenous HIV03~DISCRETE(ORDER=FORMATTED);

endogenous SUM~DISCRETE(ORDER=FORMATTED);

endogenous S306~DISCRETE(ORDER=FORMATTED);

model HIV03=HV209 HV270 V130 V501 S423;

model sum=HV206 HV270 HV108 V012 V130 V731 S122 S423;

model S306=HV206 HV209 HV270 HV108 V012 AIDSEX V130 V731 S423;

TEST 'nocorr' _rho.HIV03.sum=0,

_rho.HIV03.S306=0,

_rho.sum.S306/Wald LR;

RUN;
